# Aggregated Pattern Classification Method for improving neural disorder stage detection

**DOI:** 10.1002/brb3.3519

**Published:** 2024-08-21

**Authors:** Mohd Anjum, Sana Shahab, Shabir Ahmad, Sami Dhahbi, Taegkeun Whangbo

**Affiliations:** ^1^ Department of Computer Engineering Aligarh Muslim University Aligarh India; ^2^ Department of Business Administration College of Business Administration Princess Nourah bint Abdulrahman University Riyadh Saudi Arabia; ^3^ Department of Computer Engineering College of IT Convergence Gachon University Seongnam Republic of Korea; ^4^ Department of Computer science, College of Science and Art at Mahayil King Khalid University Muhayil Aseer Saudi Arabia

**Keywords:** classification learning, neural disorder, pattern recognition, stage classification

## Abstract

**Background:**

Neurological disorders pose a significant health challenge, and their early detection is critical for effective treatment planning and prognosis. Traditional classification of neural disorders based on causes, symptoms, developmental stage, severity, and nervous system effects has limitations. Leveraging artificial intelligence (AI) and machine learning (ML) for pattern recognition provides a potent solution to address these challenges. Therefore, this study focuses on proposing an innovative approach—the Aggregated Pattern Classification Method (APCM)—for precise identification of neural disorder stages.

**Method:**

The APCM was introduced to address prevalent issues in neural disorder detection, such as overfitting, robustness, and interoperability. This method utilizes aggregative patterns and classification learning functions to mitigate these challenges and enhance overall recognition accuracy, even in imbalanced data. The analysis involves neural images using observations from healthy individuals as a reference. Action response patterns from diverse inputs are mapped to identify similar features, establishing the disorder ratio. The stages are correlated based on available responses and associated neural data, with a preference for classification learning. This classification necessitates image and labeled data to prevent additional flaws in pattern recognition. Recognition and classification occur through multiple iterations, incorporating similar and diverse neural features. The learning process is finely tuned for minute classifications using labeled and unlabeled input data.

**Results:**

The proposed APCM demonstrates notable achievements, with high pattern recognition (15.03%) and controlled classification errors (CEs) (10.61% less). The method effectively addresses overfitting, robustness, and interoperability issues, showcasing its potential as a powerful tool for detecting neural disorders at different stages. The ability to handle imbalanced data contributes to the overall success of the algorithm.

**Conclusion:**

The APCM emerges as a promising and effective approach for identifying precise neural disorder stages. By leveraging AI and ML, the method successfully resolves key challenges in pattern recognition. The high pattern recognition and reduced CEs underscore the method's potential for clinical applications. However, it is essential to acknowledge the reliance on high‐quality neural image data, which may limit the generalizability of the approach. The proposed method allows future research to refine further and enhance its interpretability, providing valuable insights into neural disorder progression and underlying biological mechanisms.

## 1 INTRODUCTION

Neural disorder diseases affect the function of neurons in the brain and nervous system, leading to various symptoms and impairments (Lepeta et al., 2016). According to the National Library of Medicine, around 600 diseases affect the nervous system and create neurological diseases. In the United States, most people suffer due to neurological diseases, which stroke, Alzheimer and migraines are the highest impact diseases. These conditions can arise from multiple causes, such as genetic mutations, environmental factors, injuries, infections, and metabolic disorders, all of which result in the malfunctioning of neurons (Zehravi et al., 2022). These diseases impose a significant burden on both the patient's family and the healthcare system. Detecting these disorders at the earliest possible stage is crucial to slow down or halt their progression (Feigin et al., 2019). Detecting neural disorders is challenging for healthcare professionals, as many of these conditions have similar symptoms and may require a combination of different diagnostic tests and assessments (Elmatboly et al., 2020). Additionally, early detection is crucial for successful treatment, and some neural disorders may not show noticeable symptoms until significant damage has already occurred in the brain (Song et al., 2022). Therefore, developing and implementing accurate and efficient diagnostic tools and methods, such as those incorporating Artificial Intelligence (AI) and machine learning (ML), are essential for improving patient outcomes and reducing the burden on healthcare systems (Kufel et al., 2023). AI techniques effectively explore large data volumes and quickly predict necessary subsets. The prediction software analyses the set using AI techniques with pattern‐ and logic‐based algorithms. The patterns identify every small change in the dataset that maximizes overall prediction accuracy. Presently, Georgia State University researchers have developed an AI‐based tool that effectively explores the fMRI images that recognize Alzheimer's and other neurological disorders with a maximum recognition rate. Neurological disorders are typically diagnosed through the use of electroencephalography (EEG) (Abiyev et al., 2020), magnetic resonance imaging (MRI) scans, computerized tomography scans, and positron emission tomography scans (Madeira et al., 2021). Therefore, image processing techniques are widely used to detect neural disease stages and track disease progression by comparing images obtained at different time points.

Image processing techniques are important tools for the early detection of neural disorders, reducing patients’ health complexity. These techniques involve analyzing and manipulating images obtained through various imaging modalities. These imaging modalities provide a detailed view of the brain's structure and function and help identify and characterize neural disorders (Duarte et al., 2022). Image processing techniques are used to extract features from images indicative of certain disease states. For example, in Alzheimer's disease, there is a characteristic pattern of atrophy or shrinkage in specific brain regions, which are detected and quantified using MRI (Yan et al., 2022). Similarly, in Parkinson's disease, there is a loss of dopaminergic neurons in the substantia Nigra region of the brain, which can be visualized using positron emission tomography scans (Feraco et al., 2021). Implementing monitoring systems in healthcare centers is crucial for capturing accurate information related to disorders. In the case of neural disorders, monitoring systems collect and analyze data from various sources, such as imaging scans, physiological measurements, and patient behavior, to provide insights into the progression of the disease and inform treatment decisions (Knight et al., 2022). Healthcare centers offer better patient care and improve outcomes for those with neural disorders by utilizing monitoring systems. Image processing methods provide doctors and healthcare professionals more accurate and detailed information for diagnosis and treatment planning (Kim et al., 2022). The data produced by these methods can also be used for further research into neural disorders, leading to a better understanding of these conditions and developing more effective treatments.

Image pattern recognition methods are commonly used for detecting neural disorders and prediction. These methods involve the analysis of images of the brain or other neural structures using algorithms that can identify patterns or anomalies in the images that may indicate the presence of a neurological disorder (Evans et al., 2022; Quoilin et al., 2021). These methods use sophisticated algorithms and ML techniques to analyze and interpret patterns in medical images. Using these methods improves the accuracy and efficiency of the neural disorder diagnosis process. ML is widely used in detecting neural disorders and classification (Kufel et al., 2023). ML algorithms learn from the patterns and characteristics of neural disorders, making it possible to classify and diagnose different disorders accurately (Mirzaei & Adeli, 2022). For example, supervised learning algorithms are trained on labeled data to recognize patterns in medical images or EEG signals. In contrast, unsupervised learning algorithms can be used for clustering and anomaly detection. ML models can also be used for predicting disease progression and treatment outcomes. Deep learning (DL) based methods are also widely used for pattern recognition in various fields, including image processing and analysis for medical diagnosis (Anwar et al., 2018). Convolutional neural networks (CNNs) are a popular class of DL algorithms used for image classification, including in the context of neural disorder classification (Choi et al., 2020). ML and DL have shown great promise in neurology and are increasingly incorporated into modern healthcare systems for improved detection and treatment of neural disorders (Devika & Oruganti, 2021).

In recent years, a plethora of image processing, pattern recognition, and classification methods have been proposed for diagnosing neurological diseases such as epileptic seizures, Alzheimer's disease, and autism spectrum disorder (ASD) (Khan et al., 2022; Zhang et al., 2021). ASD is a neurodevelopmental disorder that affects the growth and development of the brain (Hodges et al., 2020). It is characterized by impairments in social communication and interaction and repetitive patterns of behavior and interests (White et al., 2017). ASD is typically diagnosed in early childhood and is a lifelong condition. At the same time, there is no cure for ASD; early intervention and treatment help to improve outcomes and quality of life for individuals with the disorder. The diagnosis of ASD is a complex process that generally requires a comprehensive evaluation of an individual's behavioral and cognitive characteristics (McCarty & Frye, 2020). So, this study uses MRI images to explore the patient's brain function utilizing various learning techniques. Because MRI has excellent soft tissue contrast, it may detect minor abnormalities in the brain, giving it a considerable advantage in diagnosing neural illnesses. It is worth noting because it uses nonionizing radiation, which improves patient safety and lowers the hazards that could arise from exposure to ionizing radiation. Obtaining multi‐planar pictures from different perspectives facilitates the accurate localization of lesions. Furthermore, MRI can offer functional insights, including measuring brain activity, which adds to a thorough knowledge of neurological disorders. Although image processing and ML techniques have shown promise in detecting neural disorders, there is a need for more research into their accuracy and reliability, particularly for early‐stage detection. There is also a requirement to optimize the performance of these algorithms and improve their diagnostic accuracy. In addition, traditional systems are facing difficulties during the class imbalance data which causes overfitting issues and reduces the recognition error. To mitigate these issues, this paper has the following objectives.

1. To reduce the data overfitting issues and maximize the neurological disorder recognition accuracy.
2. To introduce an Aggregated Pattern Classification Method (APCM) to identify the neural disorder stage precisely.
3. To explore the correlation between the patterns to ensure system robustness and minimize the computation complexity.
4. To demonstrate the use of classification learning in identifying different neural disorder stages and the need for labeled data for accurate pattern recognition.

The paper is divided into six sections. Section 1 introduces the challenges of detecting neural disorders and the need for accurate and efficient diagnostic methods. Section 2 discusses related work in image processing and ML for neural disorder detection. Section 3 presents the proposed APCM, which involves the analysis of patterns in medical images using ML algorithms. Section 4 outlines the classification learning process for similarity check, sequential pattern classification, and new pattern recognition. Section 5 evaluates the performance of the proposed method, and a comparison study is presented in Section 6. Finally, Section 7 concludes the paper by summarizing the findings from the proposed method.

## 2 RELATED WORKS

Several ML‐based tasks have been explored on connectome data obtained from MRI scans to improve the diagnosis of neurological disorders. The use of DL methods for brain disease diagnosis is an emerging research area, and a CNN combined with a prototype learning framework was proposed to diagnose ASD based on functional brain networks constructed from functional fMRI data (Liang et al., 2021). The proposed novel framework used traditional CNN as the basic feature extractor and multiple prototypes as a top layer to represent different categories, with a generalized prototype loss function to learn the parameters of the CNN feature extractor and prototypes jointly. Similarly, Huang et al. (2020) designed a novel graph‐based classification model using the deep belief network (GBC‐DBN) and the ASD data exchange database to identify ASD patients based on complex biomarkers from functional connectivity anomalies. The model used a graph extension of K‐nearest neighbors to select remarkable connectivity features, which were then refined by a restricted path‐based depth‐first search algorithm to reduce computational complexity. The automatic hyperparameter‐tuning technique was introduced to optimize the hyperparameters of the DBN, and the simulation experiments demonstrate the superior performance of the model. Furthermore, an existing CNN architecture U2‐Net was improved to segment the median nerve in neural images (Shao et al., 2022). The segmentation performance was evaluated using various metrics, including dice coefficients, pixel accuracy, mean intersection over union, and average Hausdorff distance. The results showed that the improved U2‐Net model had significantly better segmentation performance than the U‐Net and Res‐U‐Net models.

In recent years, researchers have been exploring the use of EEG for the early detection and diagnosis of neurodevelopmental disorders such as ASD, epileptic seizure, and attention deficit hyperactivity disorder. Saeedi et al. (2021) introduced a DL framework named depressive disorder diagnosis through CNN (DDD‐CNN) for early detection of major depressive disorder using EEG signals. The framework extracted effective brain connectivity analysis from the EEG signals and constructed an image for each individual using a combination of 16 connectivity methods. Five different DL architectures were then applied to the constructed images. The experiments show that the one‐dimensional CNN‐long–short‐term memory (LSTM) algorithm achieved the highest accuracy of 99.24% in discriminating major depression disorder patients from healthy controls. A similar type of DL model called deep convolutional autoencoder—bidirectional LSTM for epileptic seizure, which achieved an accuracy of 99.8%, classification accuracy of 99.7%, sensitivity of 99.8%, specificity and precision of 99.9%, and F1 score of 99.6% on the benchmark dataset and the dataset collected by the authors (Mir et al., 2023). EEG signals are also visualized through cortical topography, representing the electrical activity with colors. However, identifying diseases through cortical topography is challenging, so authors in de Meneses et al. (2022) compared five CNN models to recognize disease patterns in cortical topography images obtained with EEG for Parkinson's disease, depression, and bipolar disorder. SqueezeNet performed the best, achieving high accuracy, precision, recall, and F1 scores as well as having the highest AUC‐ROC values for all three diseases. Furthermore, Bouallegue et al. (2020) presented a novel dynamic filtering approach for EEG signal processing, which combined finite and infinite impulse response filters with a recurrent neural network using a gated‐recurrent unit to identify and preprocess the most informative sub‐bands for a particular neurological disorder. This approach achieves an average classification accuracy of 100% for epilepsy and 99.5% for autism using Bonn, MIT, and KAU datasets.

DL models have great potential for detecting Alzheimer's disease using MRI data at an early stage, which is crucial for effective treatment and management. Basher et al. (2021) proposed a Volumetric Feature‐Based Alzheimer's Disease Diagnosis (VFADD). This method extracts volumetric features from structural MRI data's left and right hippocampi. The proposed method combined a CNN model with a deep neural network model and used a two‐stage ensemble Hough‐CNN to localize the hippocampi automatically. The extracted volumetric features were used to train and test the classification network. The proposed approach achieved average weighted classification accuracies of 94.82% and 94.02% for the left and right hippocampi, respectively, and outperformed other methods by a certain margin in the same dataset. A physics‐informed DL framework was proposed to determine brain hemodynamics (Sarabian et al., 2022). The framework used sparse clinical measurements and one‐dimensional reduced‐order model simulations to generate physically consistent brain hemodynamic parameters with high spatiotemporal resolution. The framework was validated against in vivo velocity measurements obtained via four‐dimensional flow MRI scans.

Different types of meta‐analysis have been successfully applied to analyze neuroimaging that involves pooling results from multiple studies to provide an overall estimate of the effect size or brain regions involved in a specific phenomenon. These techniques aim to identify consistent patterns of brain activity across multiple studies, increasing the power and reliability of the findings. Inhibitory control deficits are consistently observed in several psychiatric disorders. Yan et al. (2022) aimed to identify the trans‐diagnostic convergence of neuroimaging abnormalities underlying inhibitory control across psychiatric disorders using meta‐analysis. A robust trans‐diagnostic pattern of aberrant brain activation in the frontostriatal pathways was detected. Patients showed aberrant activation in the dorsal frontal inhibitory system in cognitive inhibition while in the frontostriatal system in response inhibition across disorders. Attention deficit/hyperactivity disorder and borderline personality disorder have partially overlapped symptom profiles and are highly comorbid in adults. The meta‐analysis investigated both disorders’ shared and distinct neural patterns using multimodal functional and structural neuroimaging analysis (Pan et al., 2022). The study identified common abnormalities in the temporoparietal profile and distinct patterns in frontostriatal circuitry, which could help with better pathophysiological models and differential diagnosis. In Li and Wang (2021), the authors conducted a meta‐analysis of neuroimaging studies on patients with a major depressive disorder to identify common and distinct neural patterns in adult and youth patients during emotional processing. The study confirmed the negative processing bias in major depressive disorder patients and suggested that adult and youth patients have disturbed emotional perception, appraisal, and reactivity.

Uono et al. (2022) used voxel‐based morphometry to investigate structural neural differences between individuals with ASD and typically developing individuals in recognizing facial expressions. The proposed analysis revealed a different pattern of neural correlates of emotion recognition between ASD and typically developing individuals, suggesting that they used different processing mechanisms for recognizing others’ facial expressions. Piccardi et al. (2021) developed a behavioral and neural marker to tackle ASD in infants. Attention deficit hyperactivity disorder was also identified by a neural marker that reduced the latency in the diagnosis process. Compared with other models, the developed model tackles sensor seeking based on conditions. In Anjum et al. (2023), authors discussed the importance of early recognition of neurodegenerative diseases, which involve the progressive loss of function of neurons in the brain and spinal cord. Therefore, they introduced a syndrome‐dependent pattern recognition method for early detection and progression monitoring of these diseases. It combines observed data with previous and healthy function examination data to identify variances. The proposed method achieved high accuracy, precision, and pattern verification while reducing variance and verification time.

## 3 PROPOSED AGGREGATED PATTERN CLASSIFICATION METHOD

**FIGURE 1:**
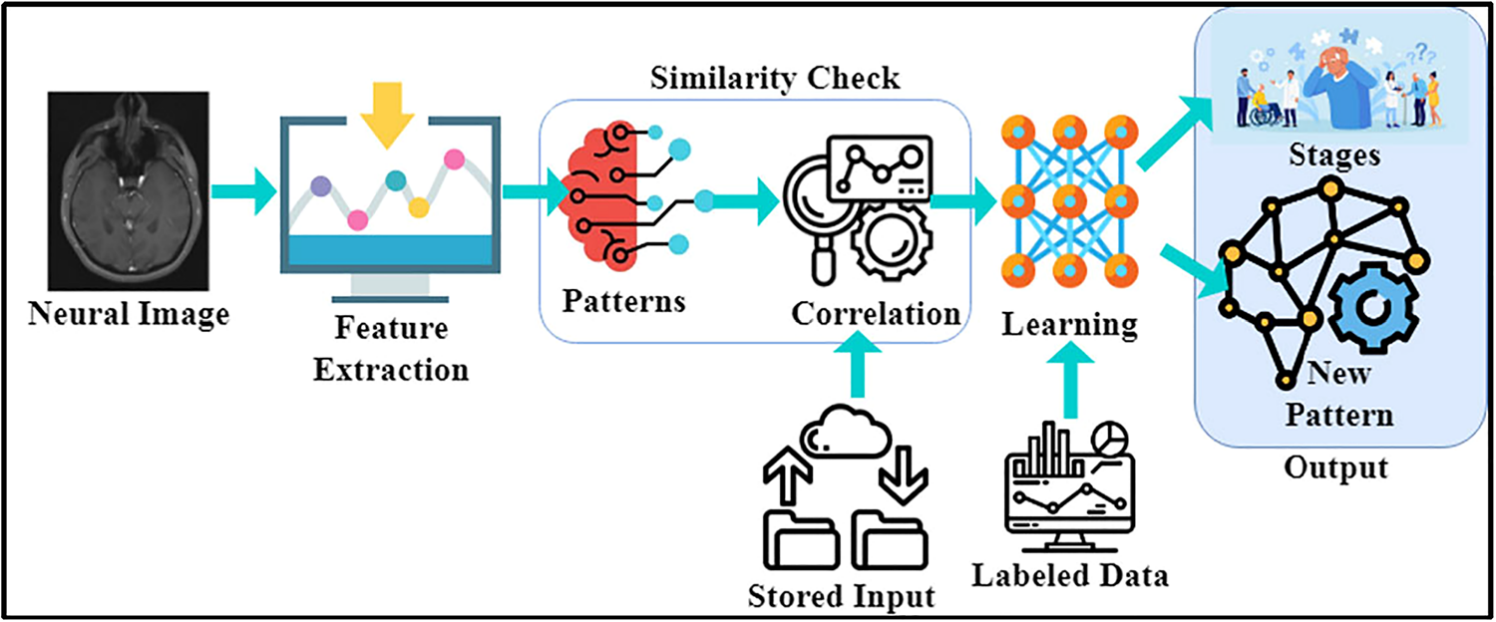
The schematic process of the proposed Aggregated Pattern Classification Method.

The design goal of APCM is to maximize the pattern recognition and classification accuracies for recognizing neurological disorders in the human brain by neural image processing to reduce the disorder ratio. The neural disorder ratio is reduced using the classification process, a variety of disorders to be controlled for identifying stage level, disorder occurrence, and its postimpact symptoms. Disorder ratio may refer to the proportion of people with neurological disorders in relation to the overall population or the proportion of the affected brain regions in relation to the total number of brain regions that are being examined. The objective is to decrease this ratio by improving the capacity to precisely diagnose and categorize neurological illnesses using APCM and other neural image processing techniques. Lowering the disorder ratio is significant because it suggests a greater knowledge of neurological disorders, their effects at various stages, and how to identify the disorder's onset and aftereffects. The APCM aims to enhance the categorization procedure, making it more dependable and accurate, which can help with neurological disease management, early identification, and intervention. In the study or application context, the method can help reduce the disorder ratio by achieving higher classification accuracies, which can aid in improving the diagnosis and understanding of these disorders. The proposed method can identify neurological disorders in all the stages for diagnosis. In practice, MRI is high resolution to identify disorders through AI and ML to secure from forging attacks and theft and improve the pattern classification method for neural disorder stage detection. APCM is a pattern recognition method designed to classify such disorders to improve the chances of recovery and treatment exposures based on textural feature extraction. The features are required from the human neural image, that is, the neurological disorder stages are detected in different time intervals. This study uses the biological markers such as gene expressions, neuroimaging features, temporal features, spatial characteristics, demographic features genetic factor, and behavioral patterns that are utilized as the features. Especially the textural features are integrated APCM to maximize the disorder identification process. The neuroimaging fed into the segmentation and voxel‐wise analysis to derive the numerical features. During the analysis, connectivity patterns, texture information, and statistical measures are derived to improve the overall disease identification process. The aggregate pattern classification method controls and reduces classification errors (CEs) in neural image processing. The factor considered in this article is pattern recognition and correlation of single patient neural disorder stage detection by image processing from the stored input. The extracted features are stored as records from the previously identified healthy person's neural observation instances. In this article, we notify the problem of accurately classifying the disorder stages individually. The different inputs from different action response patterns (RPs) are mapped for similar features for detecting the disorder ratio. The similarity features are verified based on pattern recognition, and correlation is processed to identify the disorder stage, location, and symptoms, which are analyzed using stored input. The classification function requires labeling and unlabeling to be processed across multiple iterations and analyzed using similar and diverse neural features. The process of the proposed method is explained in Figure 1.

The neural image is analyzed using a healthy person's neural observation using classification learning to identify different disorder stages and occurrences. The healthy person's neural observations are classified as labeled and unlabeled data from the different inputs. Pattern recognition and correlation processes reduce the chance of additional flaws by causing CEs. The controlled CEs are detected as an instance of recognizing neurological disorders. The proposed pattern classification method uses ML to focus on such errors through similarity checks. The first neural image processing let $NU^{\mathrm{Image}}$ indicates that the number of neural images captured from humans is recognized in an interval and processed such that the human brain behavior $BB^{i}$ is computed as

(1) BBi=NUImage−DSt×DOc×DIm×TEFX

where

(2) $$\;TE^{\mathrm{FX}}=\mathrm{argmi}n_{\mathrm{CE}}\;\sum D^{\mathrm{St}}\;\forall \;NU^{\mathrm{Image}}$$

In Equation (1), the variables $D^{\mathrm{St}}$, $D^{\mathrm{Oc}}$, and DIm illustrates the neural image classification based on disorder stage, occurrence, and its postimpact with the view of treatment or diagnosis for ease of identifying neural disorder in the human brain. The main objective of minimizing the CE is to improve the treatment exposure and increase the chances of recovery. The textural feature can be extracted for precise disorder stage identification in neural image processing. Based on the textural feature extraction, the two processes performed, namely, systematic $\;({∁}_{s})$ and accurate pattern classification $\;({∁}_{a})$. In this article, the proposed aggregate pattern classification $\;\mathrm{PC}\;={∁}_{s}\;+{∁}_{a}$ is performed to identify precise disorder stages such that ${∁}_{a}$ is detected between two $\;{∁}_{s}$. If $\;\Delta_{n}$ represents the number of disorder‐identified patients, then $\;{∁}_{a}=\left(\Delta_{n}\times \mathrm{PC}\right)\;-{∁}_{s}$ is the single‐person textural feature extraction for accurate pattern classification. Assume, $\;\mathrm{PTT}({∁}_{s})$ and $\;\mathrm{PTT}({∁}_{a})$ means the patterns of neural input image observed in t interval, and CE is identified at the time of aggregate pattern classification which is expressed as

(3) $$\mathrm{PTT}\;\left({∁}_{s}\right)=\int_{0}^{1}{∁}_{s}\;\Delta_{n}\forall \;NU^{\mathrm{Image}},\mathrm{CE}\;=\;0$$

(4) $$\mathrm{PTT}\;\left({∁}_{a}\right)=\int_{0}^{1}\frac{{∁}_{a}\Delta_{n}}{\mathrm{CE}}\;\forall \;NU^{\mathrm{Image}},\mathrm{CE}\neq 0$$

**FIGURE 2:**
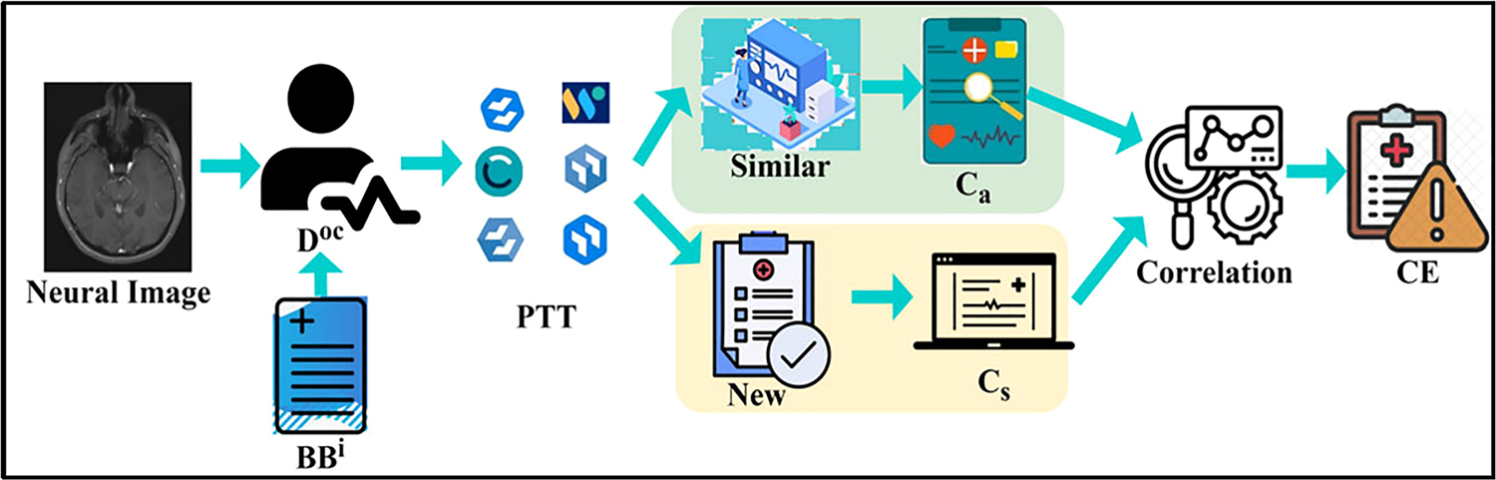
The flow diagram of the pattern classification process.

Based on Equations (3) and (4), the multiple action RPs observed from different neural image inputs are mapped with $\;NU^{\mathrm{Image}}$ for identifying precise results. The pattern classification is illustrated in Figure 2.

The input image and clinical data are jointly analyzed for pattern identification. The $D^{\mathrm{Oc}}$ is analyzed for ${∁}_{s}$ and ${∁}_{a}$ classifications for preventing error overlaps. Considering the patterns used, the training and analysis are separated for correlation. This correlation identifies $D^{\mathrm{St}}$ (from similar) and CE from $\;{∁}_{s}$ for further analysis (refer to Figure 2), and the patterns classification method is now analyzed based on stored input. So that as per Equations (3) and (4), Equation (1) is rewritten as

(5) BBi=PTT∁s=PC−DSt×DOc×DIm×∁sΔn×NUImage,CE=0PTT∁a=PC−DSt×DOc×DIm∁a+Δn×NUImage,∀e≠0

For the expanded human brain behavior instances, the sequence of systematic pattern classification is precomputed on addressing the first accurate pattern classification based on the extracted textural feature expressed as in Equation (6). This neural image is analyzed using the learning process to identify precise disorder ratios and control CEs based on similarity checks. Correlating patterns matching and similarity check are performed using the available response and the neural data associated with the image through the consecutive learning process. As per this performance, the sequence of $\;\Delta_{n}\in {∁}_{a}$ is estimated as

(6) $$\Delta_{n}\;\left({∁}_{a}\right)=\left(1-\frac{{∁}_{s}}{\mathrm{CE}}\right)\;S{T_{\mathrm{input}}}_{N}+\sum_{i\;=\;1}^{t}{\left(1-\frac{D^{\mathrm{St}}}{\Delta_{n}}\right)}^{\mathrm{PC}-1}+{\left(S{T_{\mathrm{input}}}_{N}\right)}^{t}$$

In Equation (6), the following sequence of stored input $\;S{T_{\mathrm{input}}}_{N}$ is compared with the present input image. The stored input sequence is computed using the systematic pattern classification, whereas the accurate pattern classification $\;\Delta_{n}\;\left({∁}_{a}\right)=\left(1-\frac{{∁}_{s}}{\mathrm{CE}}\right)\;S{T_{\mathrm{input}}}_{N}+\sum_{i\;=\;1}^{t}{\left(1-\frac{D^{\mathrm{St}}}{\Delta_{n}}\right)}^{\mathrm{PC}-1}+{\left(S{T_{\mathrm{input}}}_{N}\right)}^{t}$ is computed for identifying accurate disorder occurrence. Therefore, based on the process, $\;BB^{i}={∁}_{s}\;-\left[1-\Delta_{n}\left({∁}_{a}\right)\right]$ is the final estimation for identifying the disorder ratio and preventing additional flaws. The similarity check is verified based on available RPs, and correlated disorder stages (CS) are identified from the given neural input image for disorder stage detection at the first level, which is computed as

(7) $$\exists \;=\sum_{i}^{\Delta_{n}}\left(\frac{\mathrm{RP}+\mathrm{CS}}{t}\right)\;\forall NU^{\mathrm{Image}}-\mathrm{CE}$$

Equation (7) verifies the similarity of the patterns and stages observed in the sequence's systematic and accurate pattern classification. This $S{T_{\mathrm{input}}}_{N}$ is observed from the healthy person's neural processing. In this first pattern classification process, the serving inputs of ∃, $\;\mathrm{PTT}({∁}_{s})$, and $\;\mathrm{PTT}({∁}_{s})$ are used for preferred correlation using learning for detecting different disorder stages. The consecutive image processing for pattern recognition helps to detect the CE in similarity verification between systematic and accurate pattern classification. This classification learning process is briefly elaborated in the following session.

## 4 CLASSIFICATION LEARNING PROCESS FOR SIMILARITY CHECK

**FIGURE 3:**
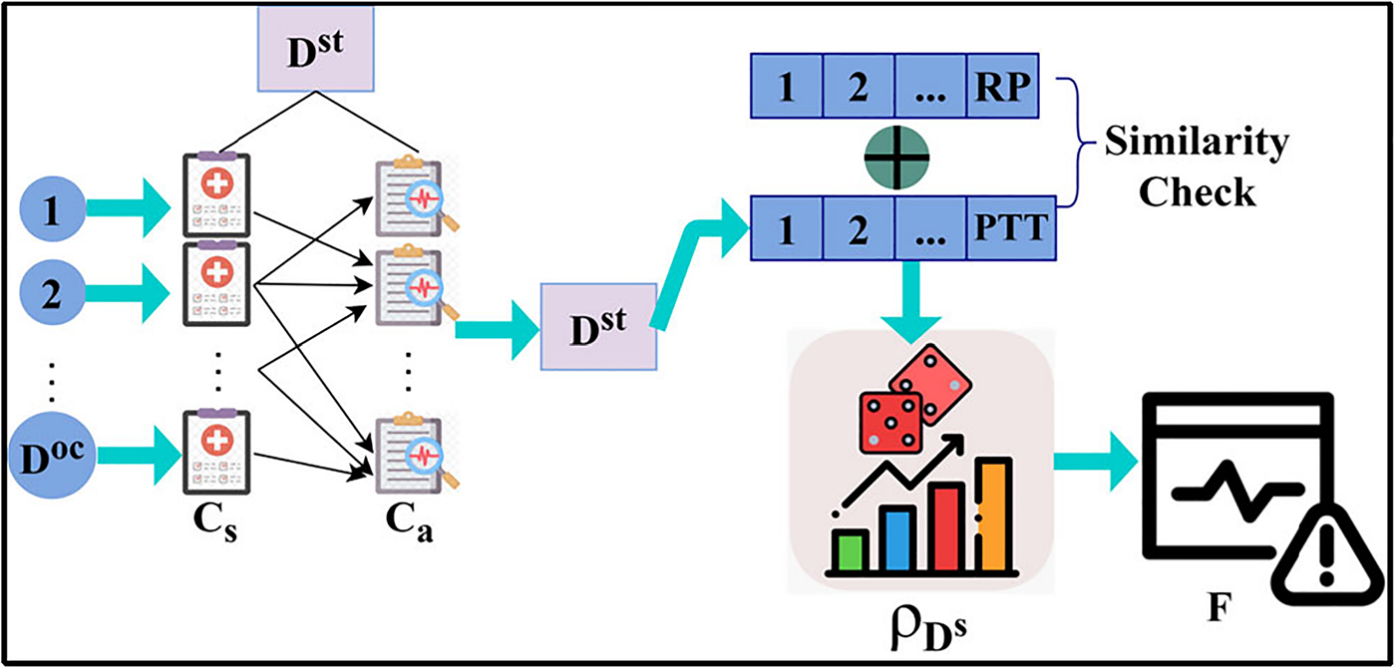
Classification learning representation for similarity check.

In the learning process, similarity verification is performed to identify the accurateness of neural disorder stage or occurrence and control CEs. As this learning depends on stored input of $NU^{\mathrm{Image}}$, observation for achieving precise results and improving the chances of recovery are high. The stages may vary for each person, though the stored input helps to identify the stages and occurrence based on the textural feature extraction and $\;\Delta_{n}\left({∁}_{a}\right)$ indifferent t intervals. In this practice, the classification learning outputs in two ways, namely, pattern recognition and sequence classification. In the pattern recognition process, different action RPs in $\;NU^{\mathrm{Image}}$ observation is mapped to identify the disorder ratio, better treatment exposure, and detection of CEs. Instead, in the sequence classification process, $\;{∁}_{a}$ and $\;{∁}_{s}$ are computed to improve the stored input of $\;NU^{\mathrm{Image}}$. The neural input image performs both pattern recognition and sequence classification for given $\;NU^{\mathrm{Image}}$ in t instances. The classification learning representation for similarity check is presented in Figure 3.

The $\;D^{\mathrm{St}}\;\forall \;D^{\mathrm{Oc}}$ is segregated as $\;{∁}_{s}$ and ${∁}_{a}$ and the $\mathrm{PTT}\;\in {∁}_{a}$ are verified for similarity with RP. Based on the available patterns, the RP generates $BB^{i}$ data $\;\forall \;\Delta_{n}\left({∁}_{a}\right)$. Using the $\rho_{D^{S}}$ that occurs in the mediate ∃, and the F is extracted. If the extracted F is valid for the analysis, error suppression is high; contrarily, the fewer chances increase CE possibility (refer to Figure 3). The approximation of $\;NU^{\mathrm{Image}}\in t$ is mapped under $\;{∁}_{a}$ and $\;{∁}_{s}$ based on the disorder occurrence and stage. The classification of the patterns is correlated with identifying different disorder stages through ML. Later, based on the pattern classification, the chances of recovery and treatment exposure are the augmenting factor. For the sequence, the neural disorder stage and new pattern identification are the prevailing instances trained using labeled and unlabeled data. The different inputs describe the similar and diverse neural features used for performing multiple iterations in the following manner.

### 4.1 Sequential pattern classification

In this process, the learning is trained for minute classifications from the different neural image inputs based on labeled data $\;L_{\mathrm{data}}$, which are the deciding metric. The probability of identifying disorder stages $\;(\rho_{D^{s}})$ is expressed as

(8) $$\rho_{D^{s}}={\left(\;1-F\right)}^{\Delta_{n}-1}\;\forall \;\Delta_{n}\in t$$

where

(9) $$\;F\;=\left(1-\frac{\exists \in \Delta_{n}}{\exists \in t}\right)\;$$

where the variable F denotes additional flaws in sequential pattern classification for identifying precise disorder stages using input images such that there are no unlabeled data, based on the labeled data, easily identify the person and their disorder stage using classification learning. Therefore, treatment is recommended Recomd(T) for the neural disorder, an identified person is expressed as

(10) $$\mathrm{Recomd}\;\left(T\right)=\frac{1}{\left|\mathrm{RP}-\mathrm{CS}\right|}\;{\left(\rho_{D^{s}}\right)}_{\Delta_{n}}\;\forall \;\Delta_{n}\in t$$

**FIGURE 4:**
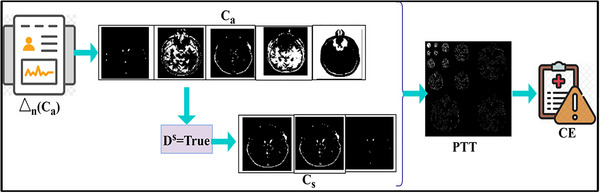
The schematic diagram of sequential PTT classification.

However, the recommendation for diagnosing the neural disorder is achieved for both recognition and classification performance ensuring available RPs from various inputs. The correlating process of stages is identified to reduce the adversarial impact and flaws using the condition $\mathrm{PTT}({∁}_{s})$ and $\;\mathrm{PTT}({∁}_{a})$. The sequential pattern classification process is illustrated in Figure 4.

The above sequential classification relies on a similarity check performed using $\;\rho_{D^{S}}$. If this probability is true, the flaw occurrence is high and therefore recommended for the observed $D^{\mathrm{St}}$ provided. Considering the $\Delta_{n}\left({∁}_{a}\right),$ the extraction for ${∁}_{s}$ is also performed such that RP patterns are extracted. Therefore, the next sequence is validated for CS (refer to Figure 4). The disorder stages are recognized using sequential pattern classification and labeled data. Therefore, comparing the labeled data and disorder stage is less to satisfy Equation (1). The contrary output, for instance, identifies additional flaws and the classification time increases, resulting in CEs.

### 4.2 New patterns recognition

In the new pattern recognition process, the available response and the associated neural data with the image vary; hence, the sequential pattern classification is unlabeled. The additional flaws and CEs are increased along with the required image and unlabeled data. To reduce these features, using similarity verification results from the stored input. The probability of new pattern recognition $\;(\rho_{NP^{r}})$ is computed as

(11) $$\rho_{NP^{r}}=\frac{\rho_{D^{s}}.\;\mathrm{Recomd}\left(T\right)\left[\left(\mathrm{RP}-\mathrm{CS}\right)F-\left(\frac{\mathrm{RP}-\mathrm{CS}}{\Delta_{n}}\right)\frac{\mathrm{CE}}{t}\right]\varphi \left(A\right)}{\varphi \left(A\right)\times \Delta_{n}}\;$$

where

(12) $$\;\left.\begin{array}{c} \varphi \;\left(A\right)=\;\int_{0}^{t}PC^{t-1}{\left(1-\exists \right)}^{t-1}\;dt\\ and\\ \varphi \left(A\right)\in Recomd\;\left(T\right)=\int_{1}^{\mathrm{RP}}PC^{t-1}\frac{Ft}{\Delta_{n}}\;{\left(1-\rho_{D^{s}}\right)}^{t-1}\;dt\; \end{array}\right\}$$

where the variable φ(A) indicates that the new pattern recognition from the input $\;NU^{\mathrm{Image}}$ is compared using stored input. For all the pattern recognition checking, the unlabeled data are identified in the instance of the problem or flaw overflow. Therefore, if the new patterns are identified from the input image, those neural images are classified based on stages, occurrence, and impact with the view of diagnosis and then labeled the data as per the stages, thereby increasing the classification accuracy.

**FIGURE 5:**
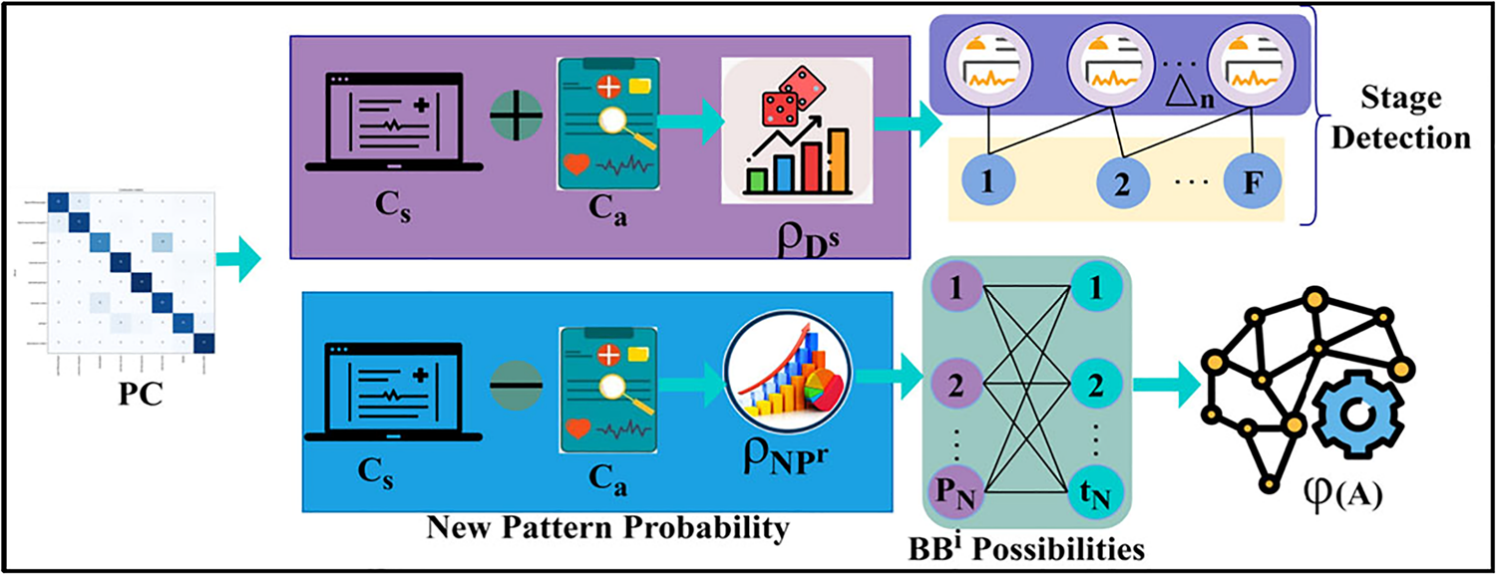
The process of new pattern recognition.

From the analysis of pattern recognition and sequential classification identification, neurological disorders can be detected based on labeled and unlabeled data from the different inputs to prevent flaws and CEs. The neurological disorder is addressable using classification learning to control the post impacts and errors through recurrent analysis. The classification learning process is performed for mapping both recognition and classification based on the conditions CE≠0, ${∁}_{a}=\;\left(\Delta_{n}-\mathrm{CE}\right)\;{∁}_{s}$, and $\;\;NU^{\mathrm{Image}}$. If the similarity check is verified with the available features in the classification time, it is 1; otherwise, 0. The output of the disorder stages identification generates precise results, whereas the new pattern recognition extracts the features and similarity check and then generates output with CE≠0. As per the learning process, the two outputs are estimated for identifying neurological disorders persons based on their behavior. Therefore, the output is required from the different inputs and pattern classification time interval t. Based on the practices, the above mapping process with $\;L_{\mathrm{data}}$ serves as an input for post detection of neurological disorders persons through healthy person brain behavior, and disordered person brain behavior is expressed as

(13) $$\left.\begin{array}{c} \;P_{1}={∁}_{a_{1}}\;t_{1}+\exists_{1}-CE_{1}\\ \;P_{2}={∁}_{a_{2}}\;t_{2}+\exists_{2}-CE_{2}\\ \begin{array}{c} \;P_{3}={∁}_{a_{3}}\;t_{3}+\exists_{3}-CE_{3}\;\\ \vdots \\ \;P_{N}={∁}_{a_{t}}\;t_{t}+\exists_{t}-CE_{t} \end{array} \end{array}\right\}$$

where the variable $P_{1}\;to\;P_{N}$ represents the group of neurological disorder‐identified persons recognized through similarity analysis. The new pattern recognition process is briefed in Figure 5.

The new pattern is recognized from the ${∁}_{s}$ leaving out CE that reduces the $D^{\mathrm{St}}$ classification from F. Therefore the $F\;\forall \Delta_{n}$ (alternating) is identified for the existing patterns. Different from this, the $BB^{i}$ possibilities for $P_{N}\times t_{N}\;\forall \exists_{N}$ are used for identifying φ(A) with more recommendations and training (refer to Figure 5). This observation is processed until the accurate pattern classification is valid. Instead, CE continuously deviates both recognition and classification until the similarity check is performed for diverse or similar features. From the feature extraction and stored input, the learning is trained to update new pattern recognition based on similarity checks, increasing the chances of recovery and treatment exposure to reduce the CE and additional flaws in different time intervals.

## 5 PERFORMANCE ASSESSMENT

**FIGURE 6:**
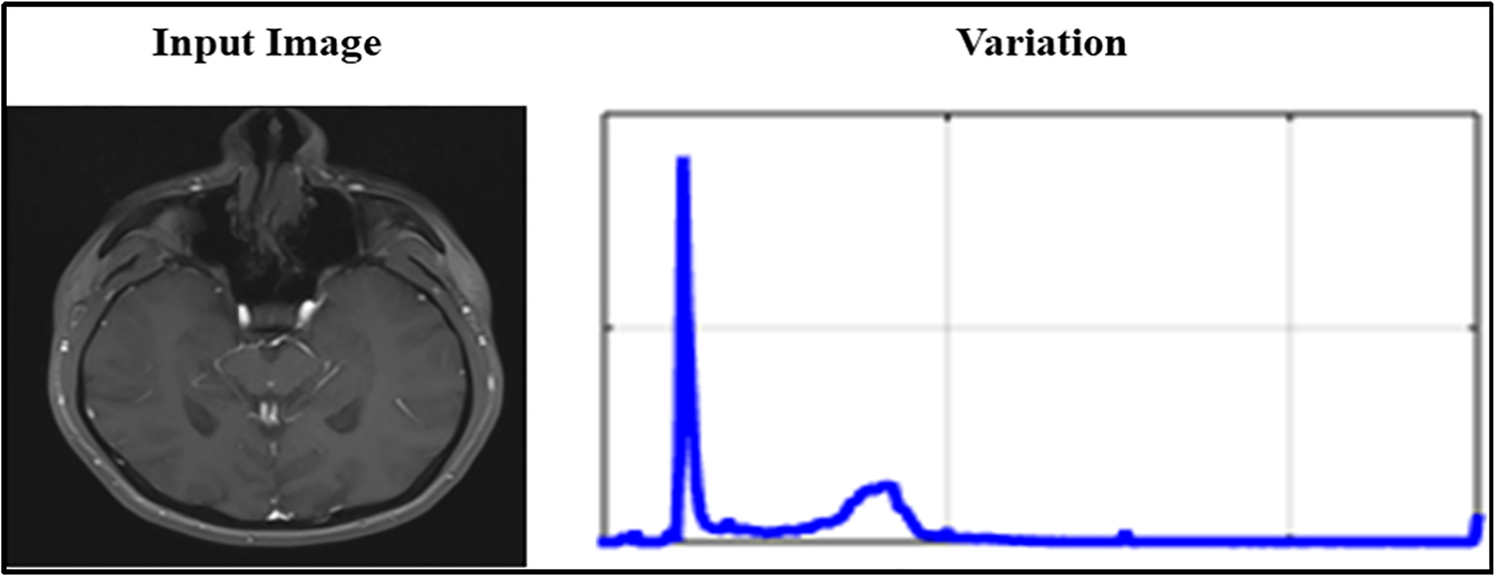
$NU^{\mathrm{Image}}$ and $D^{\mathrm{Oc}}$ variation.

The proposed method's performance is analyzed using the Autism Brain Imaging Data (Di Martino et al., 2014). This dataset records information on 1112 in which 539 individuals collected from ASD and 573 from controls. Each subject having 26 attributes that effectively utilized to detect the neurological disorder. The image frequency varies between 0.01 and 0.1 Hz, handled by Gaussian filtering. From this input, the features are extracted from 2 to 30, and a total of 130 classifications are performed. For an $NU^{\mathrm{Image}}$, the variation due to $D^{\mathrm{Oc}}$ is illustrated in Figure 6. This variation generates a minimum of two patterns and a maximum of N patterns (as presented in Figure 4).

**FIGURE 7:**
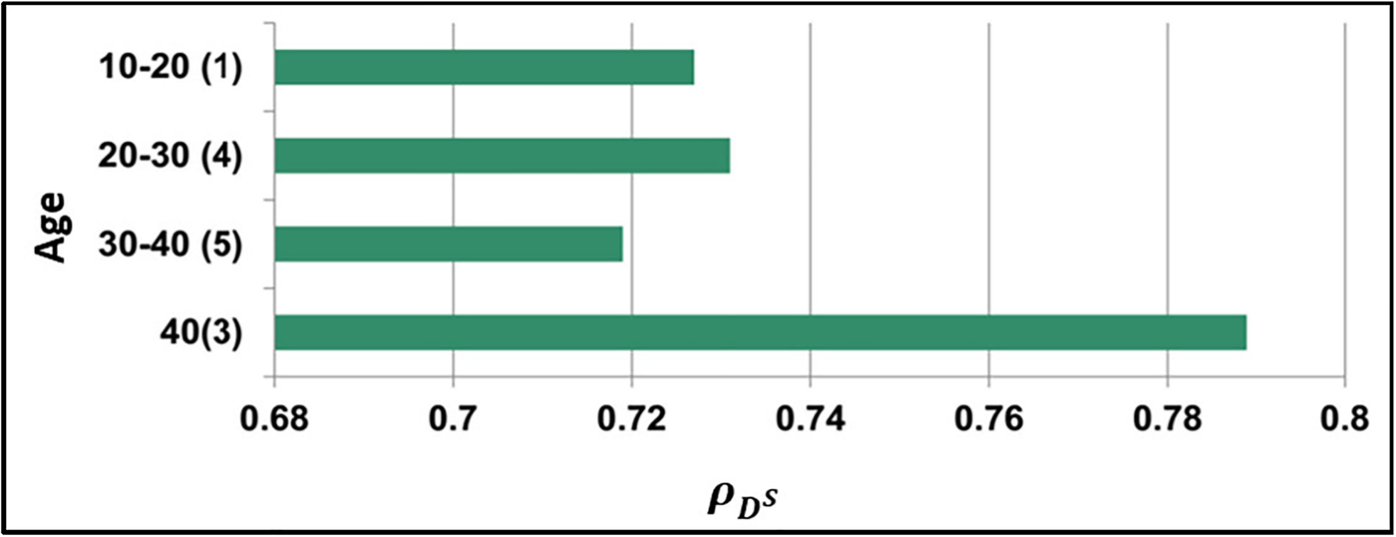
$\rho_{D^{S}}$ for different ages among 13 subjects.

The $\;D^{\mathrm{Oc}}$ illustrates the need for classification until $\;\rho_{NP^{r}}\;$ is false. This is required for validating CS with the given data. From the accumulated medical input, the $\rho_{D^{s}}\;$ for different ages is analyzed in Figure 7.

**FIGURE 8:**
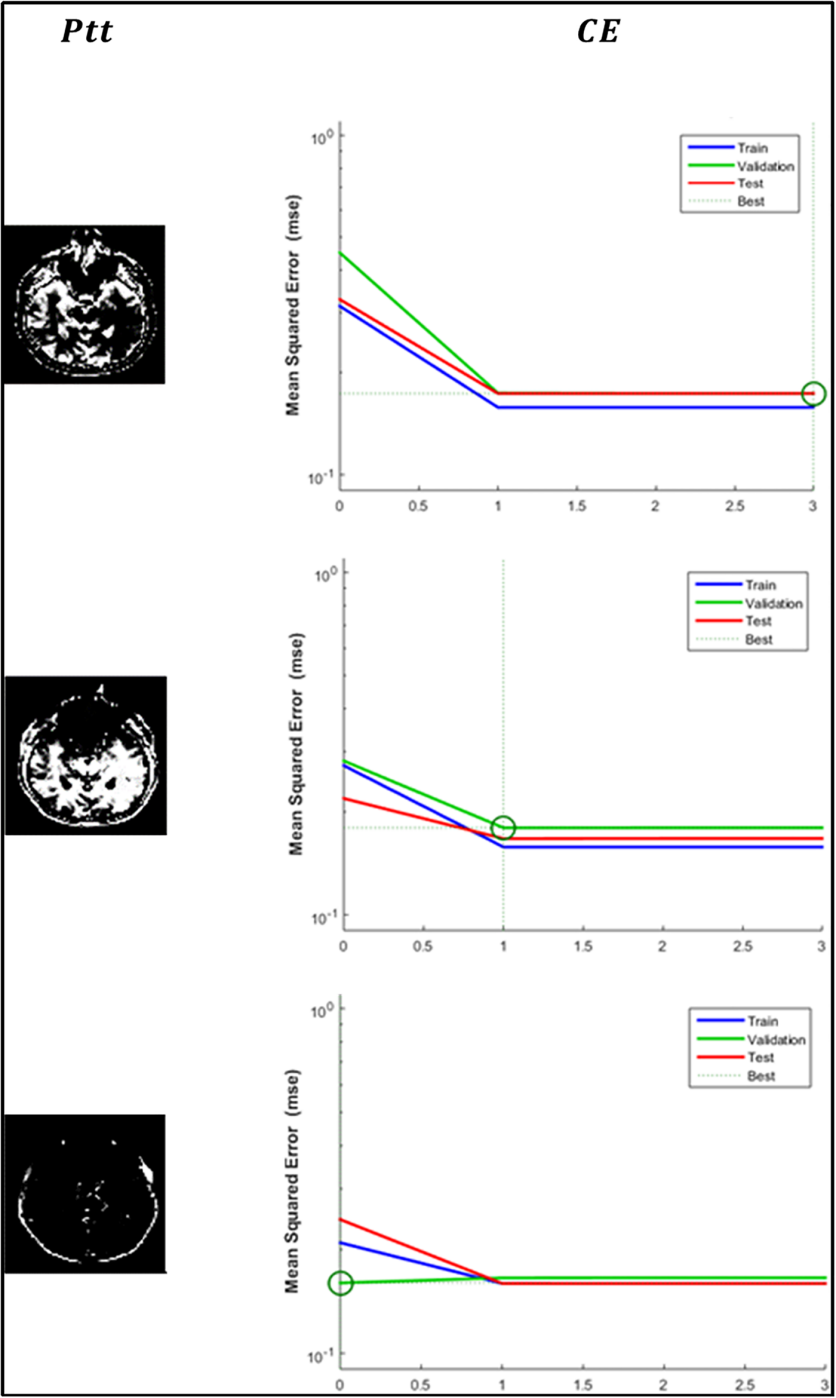
Error analysis.

The age variations generate ${∁}_{s}$ and $\;{∁}_{a}$ results for which $\;\rho_{D^{s}}\;$ and $\;\rho_{NP^{r}}\;$ are concurrently analyzed. The classification is based on RP and PTT (as in Figure 4), and the identification of $\rho_{D^{S}}$ is performed. This disorder turns acute for people with mental illness such that the flaws are invariable. Therefore, the flaw (error) detection is to be précised under the varying $D^{\mathrm{Oc}}$ for which the analysis is presented in Figure 8.

**FIGURE 9:**
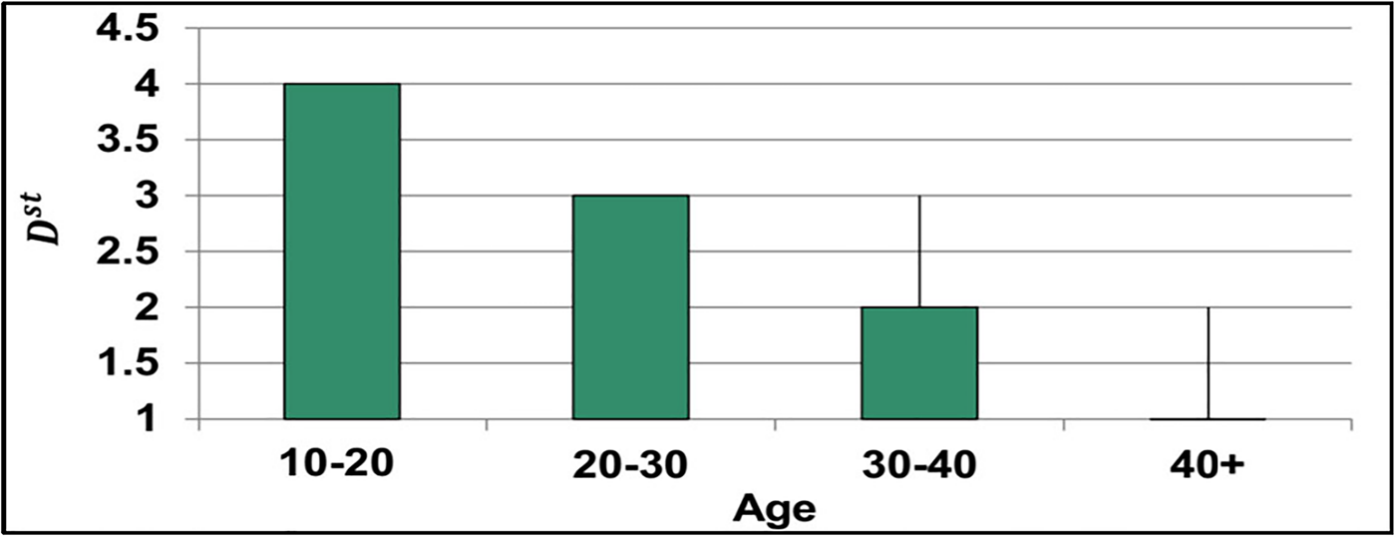
$D^{\mathrm{St}}$ classification for different ages.

The above error analysis is presented for different classification epochs. In the epoch's variation, the error reduction is focused on promoting $\;{∁}_{s}+\mathrm{CE}$ and ${∁}_{S}-\mathrm{CE}$ across various φ(A). The training and validation are performed by benchmarking the most affordable solution. Therefore, the epochs in the classification $\forall \;P_{N}\times t_{N}$ are varied until the best afford is achieved. The dividing classifications based on PTT and reduced CE maximize the accuracy through feature detection. Posting this analysis, the stage classification among different ages is analyzed in Figure 9.

The classification of $D^{\mathrm{St}}$ requires $BB^{i}$ and DIm for ${∁}_{a}$ and $\;{∁}_{s}$ validations. This requires multiple classifications for preventing F merging as CE; the abrupt classifications are required ∀(t+1). This process ∃ is executed for $\Delta_{n}\left({∁}_{a}\right)$ before the ${∁}_{s}$ is estimated. Therefore, $\;P_{1}$ to $P_{N}$ reinitiates the further classification for preventing new patterns (refer to Figure 9).

## 6 COMPARISON STUDY

The comparison study is performed using pattern recognition ratio, classification accuracy ratio, CEs, classification time, and feature identification. To provide the effectiveness of the proposed method, a comparative study is performed with GBC‐DBN (Huang et al., 2020), DDD‐CNN (Saeedi et al., 2021), and VFADD (Basher et al., 2021) methods.

### 6.1 Pattern recognition

**FIGURE 10:**
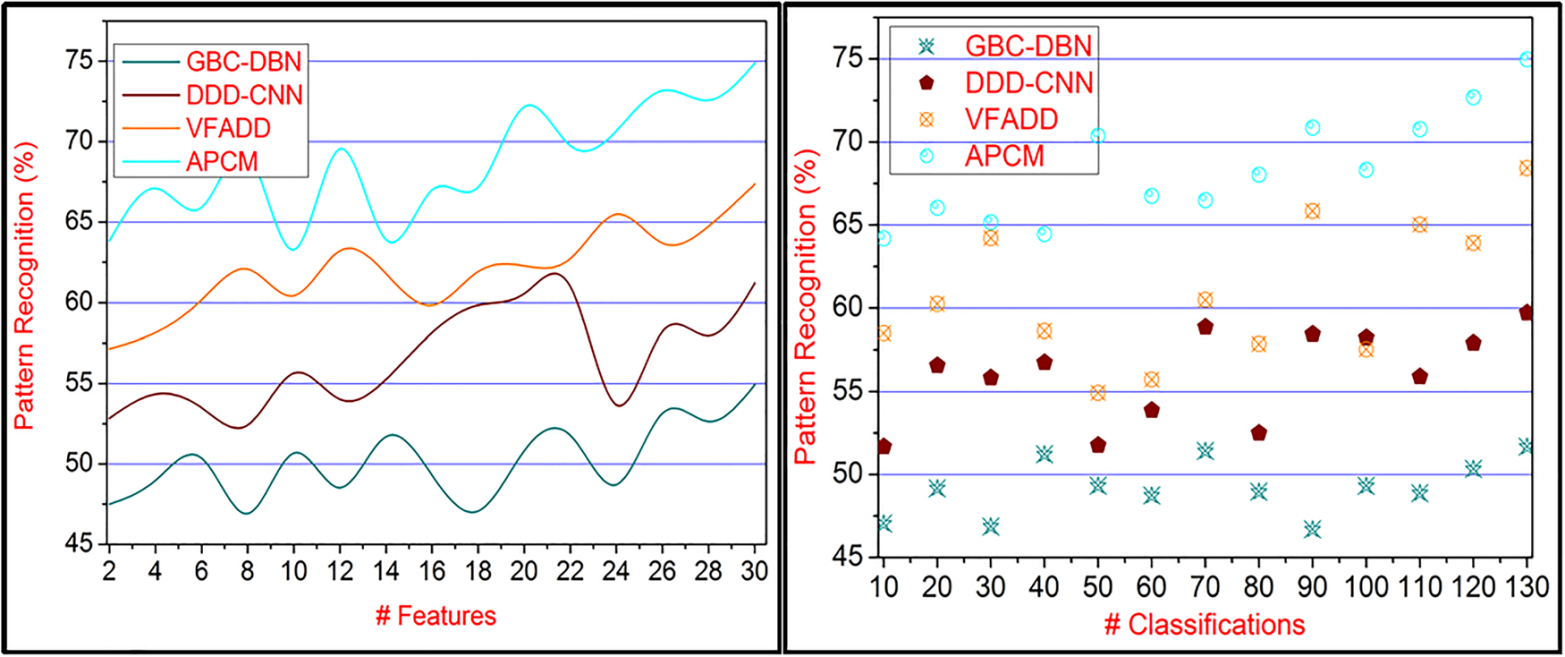
Pattern recognition comparisons for varying features and classifications.

Figure 10 represents the textural feature extraction for human neural image processing based on brain behavior and activities. In the proposed method, pattern recognition is maximized by identifying neurological disorder persons by systematic and accurate pattern classification. The initial occurrence of CE is based on different action RPs for all the disorder‐detected sequences. For this sequence, pattern recognition is estimated through labeled and unlabeled data, whereas minute classification achieves some errors due to classifying such disorders in their early stages. Therefore, pattern recognition is modeled to identify similar and diverse neural features; pattern recognition is better finding. In addition to the occurrence of sequence classification for the instances, ${∁}_{s}$ belongs to $\;{∁}_{a}$ it identifies the correlated stages in an input image. Therefore, the number of pattern recognition leveraging instances is high in the proposed method in a considerable manner. This disorder identification based on accurate pattern classification is supported by the ML process using stored inputs for processing classification and pattern matching. Detecting such disorders maximizes pattern recognition such that $\;\mathrm{PC}\;={∁}_{s}\;+{∁}_{a}$ is the highest pattern recognition‐achieving factor in the proposed method.

### 6.2 Classification accuracy

**FIGURE 11:**
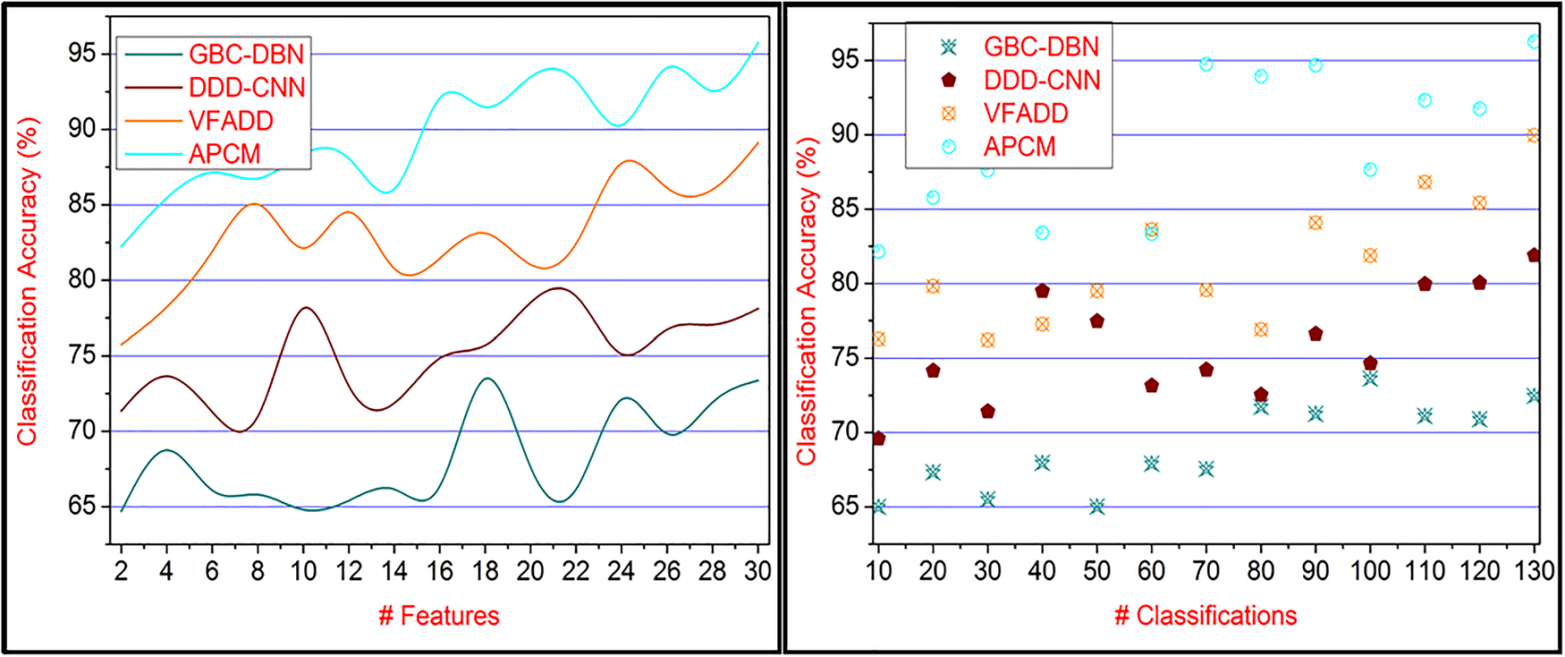
Classification accuracy comparisons for different numbers of features and classifications.

In Figure 11, AI and pattern recognition automatically detect the neurological disorders of persons based on image processing and classification for precise results. The textural features are extracted from mapping the different inputs in different time intervals. The features and brain behavior are observed from disordered persons, and similarity checks with healthy persons’ neural observations are detected for accurate identification and stages. The classification learning outputs are in continuous stages of identification with labeled data and new pattern recognition with unlabeled data. Based on the available response and the neural data associated with the input image computations, the extracted features satisfy both the condition of $\mathrm{PTT}({∁}_{s})$ and $\;\mathrm{PTT}({∁}_{a})$ for identifying the precise disorder stage and occurrence. The additional flaws and CEs are identified using the classification learning process and achieve successive disorder person identification from the different inputs, preventing misdetection. Both pattern recognition and correlation satisfy high classification accuracy in detecting neurological disorders based on brain behavior and activities of the persons, and the flaws are prevented, achieving high classification accuracy using the proposed method.

### 6.3 Classification errors

**FIGURE 12:**
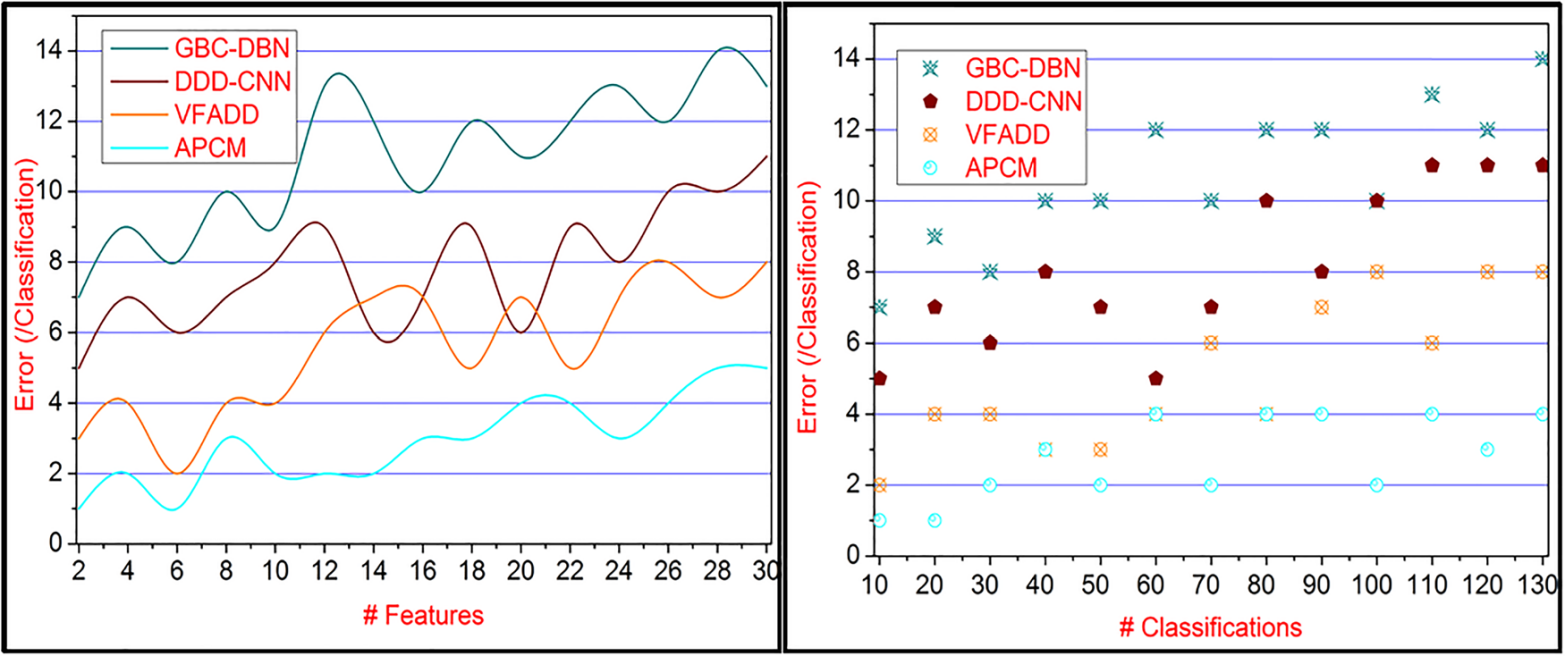
Classification errors comparison for various features and classifications.

In Figure 12, the CE identified in the proposed pattern classification method is considerably less than the observation and mapping of pattern recognition and correlation. Based on the brain, behavior detection at the first stage is used for computing the similarity verification with stored input for the first‐person output. This observation using the classification learning and stored input of a healthy person's brain behavior observation helps to map the instances for accurate pattern classification and stage detection. After this classification, pattern recognition and classification‐based similarity verification are performed for the individuals to control classification time and errors. The difference in textural features is identified using systematic, and accurate pattern classification is the first computable measure with labeled data and CE. This CE is updated using the learning training for the sequential detection states of stages using correlation from the different inputs at the end of similar feature identification. The instances achieve the diverse features and classify such disorders wherein the new patterns recognition is verified, and CE is observed from the sequence due to minute classification through unlabeled data. This classification helps to reduce the postimpact with the view of diagnosis in all the instances across different inputs, reducing CE in this proposed method.

### 6.4 Classification time

**FIGURE 13:**
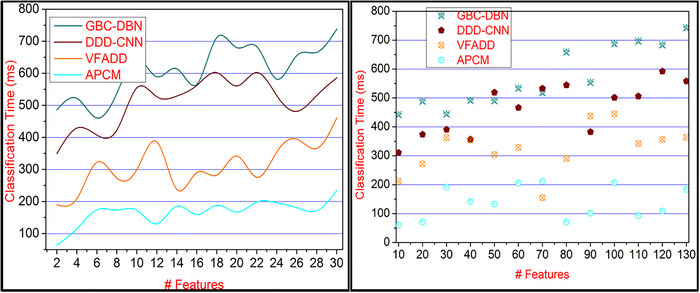
Classification time comparisons for varying features and classification.

The CE and time identification in pattern classification from the different inputs for a neurological disorder person's behavior detection through textural feature extraction are illustrated in Figure 13. This proposed method satisfies less classification time by computing the pattern recognition and correlation based on the disorder stage, occurrence, and its post impact, which are accurately observed for recovering from this disorder. In this neurological disorder stage detection based on a correlation process, the condition $\sum_{i}^{\Delta_{n}}\left(\frac{\mathrm{RP}+\mathrm{CS}}{t}\right)\forall NU^{\mathrm{Image}}-\mathrm{CE}$ is estimated for precise results. The CEs and additional flaws are mitigated depending on the pattern classification until diverse feature detection wherein the different action RPs observations are preceded using Equations (4–8) computations. In this proposed method, both pattern recognition and classification are processed to identify the disorder ratio. Based on this sequential pattern recognition, the classification time is less compared to the other factors in this article.

### 6.5 Feature identification

**FIGURE 14:**
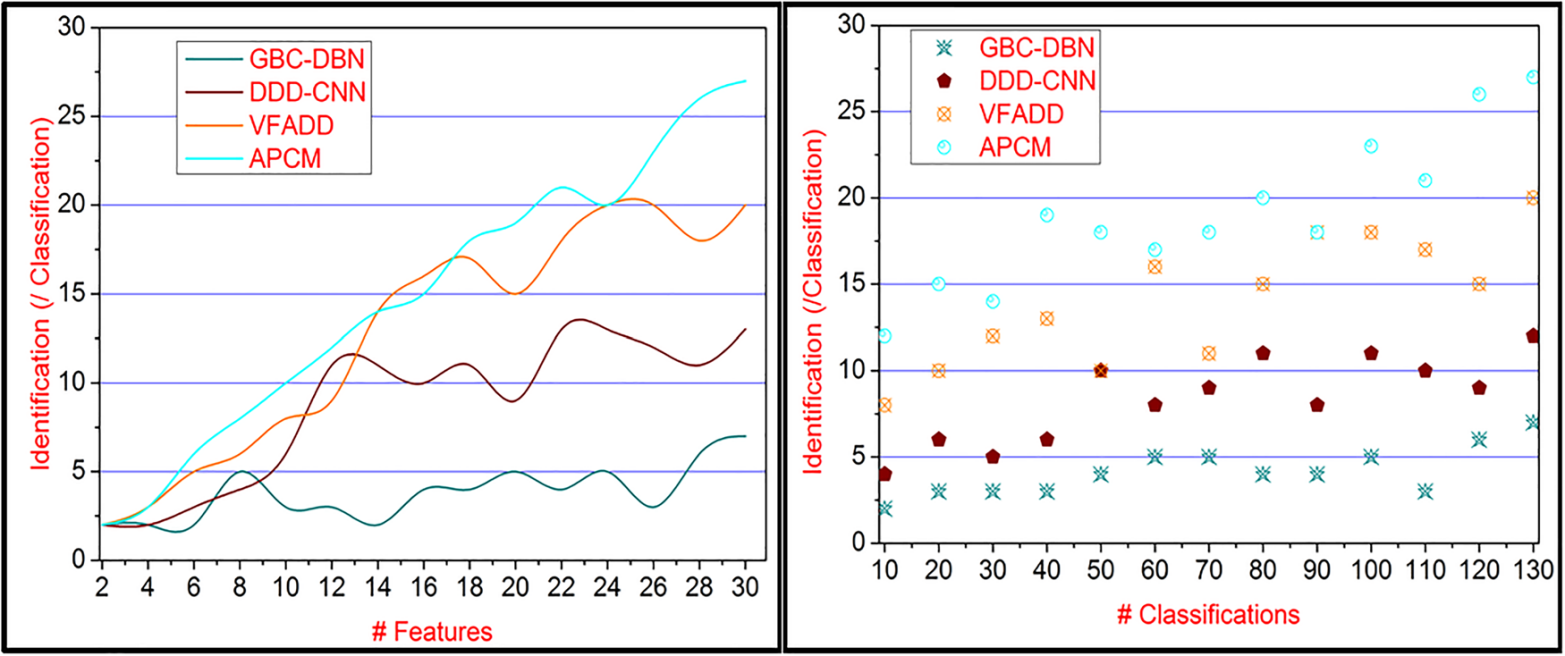
Feature identification comparisons for varying features and classifications.

Pattern recognition based on textural feature extraction of a disordered person is observed for identifying the accurate stage and occurrence depicted in Figure 14. This proposed method satisfies fewer CEs and flaws by computing the similarity verification using a learning process. In this sequential process for neurological disorder detection through image processing in different time intervals, $S{T_{\mathrm{input}}}_{N}$ and $L_{\mathrm{data}}$ are computed until identifies diverse features from the available response. The classification requires image and labeled data to reduce additional flaws during pattern recognition from the different inputs for the similarity feature analysis. The CE identified for identifying the disorder ratio for computing the pattern recognition based on available response and the neural data associated with the input image easily identifies the disorder stage using classification learning. Therefore, pattern recognition and classification are computed for similarity analysis. Hence, this proposed method identifies the disorder stage using similar and diverse neural features computed across multiple iterations, and feature identifications are high.

## 7 CONCLUSION

This article has introduced and discussed APCM to identify neural disorder diseases using image data. The proposed method extracts feature from the input images and analyses their patterns. The identified patterns are correlated with the previous identifications and extracted features. In the identification process, classification learning is exploited for segregating sequential and flaw‐based patterns. Such identified patterns are used for training the learning paradigm to prevent errors. The verification is performed using a similarity metric to identify further accurate and systematic features for the sequential patterns extracted. The unlabeled data associated with the inputs are validated using disorder stages and their corresponding identification. Based on the classification, output recommendations are provided. The new patterns associated with the accurate similarity measure are dissolved in the left‐out classifications. Therefore, due to unclassified patterns, the proposed method deprives errors and classification time. Under the varying classifications, this proposed method has achieved 15.03% high pattern recognition, 14.8% high classification accuracy, 10.61% less error, 11.16% less classification time, and 8.64% high identification. However, the system effectively classifies the diseases, availability of high‐quality neural image data, which can be limited and affect the generalizability of the method. Future research could focus on enhancing the interpretability of the aggregated patterns to gain insights into the neural disorder progression and underlying biological mechanisms.

## AUTHOR CONTRIBUTIONS

**Mohd Anjum**: Conceptualization; methodology; formal analysis; writing—original draft. **Sana Shahab**: Conceptualization; methodology; data curation; validation; writing—original draft; writing—review and editing. **Shabir Ahmad**: Methodology; formal analysis; data curation; visualization; validation; writing—review and editing. **Sami Dhahbi**: Funding acquisition; validation; visualization; writing—review and editing. **Taegkeun Whangbo**: Formal analysis; visualization; validation; writing—review and editing; funding acquisition.

## CONFLICT OF INTEREST STATEMENT

No potential conflicts of interest relevant to this article was reported.

### PEER REVIEW

The peer review history for this article is available at https://publons.com/publon/10.1002/brb3.3519

## Data Availability

The performance of proposed model was evaluated using the Autism Brain Imaging Data which is freely available in public domain at https://www.nature.com/articles/mp201378, DOI: https://doi.org/10.1038/mp.2013.78.
